# Neural basis of negativity bias in the perception of ambiguous facial expression

**DOI:** 10.1038/s41598-017-00502-3

**Published:** 2017-03-24

**Authors:** Takehito Ito, Keita Yokokawa, Noriaki Yahata, Ayako Isato, Tetsuya Suhara, Makiko Yamada

**Affiliations:** 1 Department of Functional Brain Imaging Research, National Institute of Radiological Sciences, National Institutes for Quantum and Radiological Science and Technology, 4-9-1 Anagawa, Inage-ku, Chiba 263-8555 Japan; 2 Department of Molecular Imaging and Theranostics, National Institute of Radiological Sciences, National Institutes for Quantum and Radiological Science and Technology, 4-9-1 Anagawa, Inage-ku, Chiba 263-8555 Japan; 3 Group of Quantum and Cellular Systems Biology, QST Advanced Study Laboratory, National Institutes for Quantum and Radiological Science and Technology, 4-9-1 Anagawa, Inage-ku, Chiba 263-8555 Japan

## Abstract

Negativity bias, which describes the tendency to interpret ambiguous stimuli or events as negative, is often observed in patients with depression and may prevent psychological well-being. Here, we used ambiguous facial stimuli, with negative (sad) and positive (happy) emotions simultaneously accessible, to examine neural activation during perceptual decision-making in healthy participants. The negativity bias was positively correlated with the activity of the bilateral pregenual anterior cingulate cortex (pgACC) when ambiguous faces were perceived as sad versus happy. Additionally, the strength of the functional connectivity between the bilateral pgACC and the right dorsal ACC (dACC)/right thalamus was positively correlated with hopelessness, one of the core characteristics of depression. Given the role of the pgACC as a major site of depressive affect and the roles of the dACC and thalamus in conflict monitoring and vigilance, respectively, our results reveal valid and important neuroanatomical correlates of the association between negativity bias and hopelessness in the healthy individuals.

## Introduction

Emotional facial expressions are an important component of social communication. The processing of facial expressions is a fundamental step in social functioning, guiding adequate social interaction^1^. The ability to correctly assess emotional information extracted from facial expressions modulates social interaction and is crucial to the development of interpersonal skills and adaptive behavior. Interpersonal dysfunction (e.g., interpersonal conflict or negative cognitions surrounding interpersonal interactions) contributes to both the development and maintenance of depression^2^, which may be caused by negativity biases in the interpretation of emotional expressions^3^.

Many studies have used computerized morphing procedures to generate systematic ambiguity in facial expressions to examine biases in emotion identification and classification^4–10^. Although many of these studies used blended faces combining neutral and emotional expressions, the results may reflect perceptual sensitivity to emotional intensities rather than interpretative biases^11^. It has been suggested that ambiguous faces that contain conflicting emotional information (e.g., morphing sad and happy expressions) may be more effective for investigating interpretation biases^12^. Indeed, real-life situations are complex, and we often encounter ambiguous emotional expressions that can be perceived as either negative or positive emotions. When a person evaluates such ambiguous stimuli, one emotion may have disproportionately more influence than the other emotion, producing affective asymmetry. Several behavioral studies have shown that depression is characterized by the tendency to interpret ambiguous stimuli or events pessimistically; this negativity bias has been reported to be particularly prominent in the processing of emotional faces, in particular sad facial expressions^13, 14^.

Brain imaging techniques such as functional magnetic resonance imaging (fMRI) have already made substantial contributions to the understanding of how faces and facial expressions are processed in both clinical and non-clinical participants^15–17^. The pregenual anterior cingulate cortex (pgACC) and its connected circuitry have been heavily implicated in emotion function and in the genesis of depression^18–22^. Given its anatomical connections to subcortical and cortical structures, the pgACC is thought to lie at the interface between affective and cognitive processing, such that aberrant functioning in this region leads to impaired emotional regulation^23, 24^. In particular, in patients with depression, the perception of sad expressions is associated with abnormal hyperactivity in the pgACC^24, 25^. It has accordingly been hypothesized that negativity bias may be associated with hyperactivity of the pgACC^26, 27^. Several studies have examined stimulus-driven brain activity^28, 29^ and neural responses involved in negative emotion recognition from neutral and ambiguous stimuli^24, 25^; however, the neural substrates of the interpretative bias that weighs negative emotion over positive emotion remain unknown both in depressed patients and in healthy subjects. Thus, before investigating patients, it is important to understand the neural mechanisms of negativity bias in the healthy individuals.

**Figure 1:**
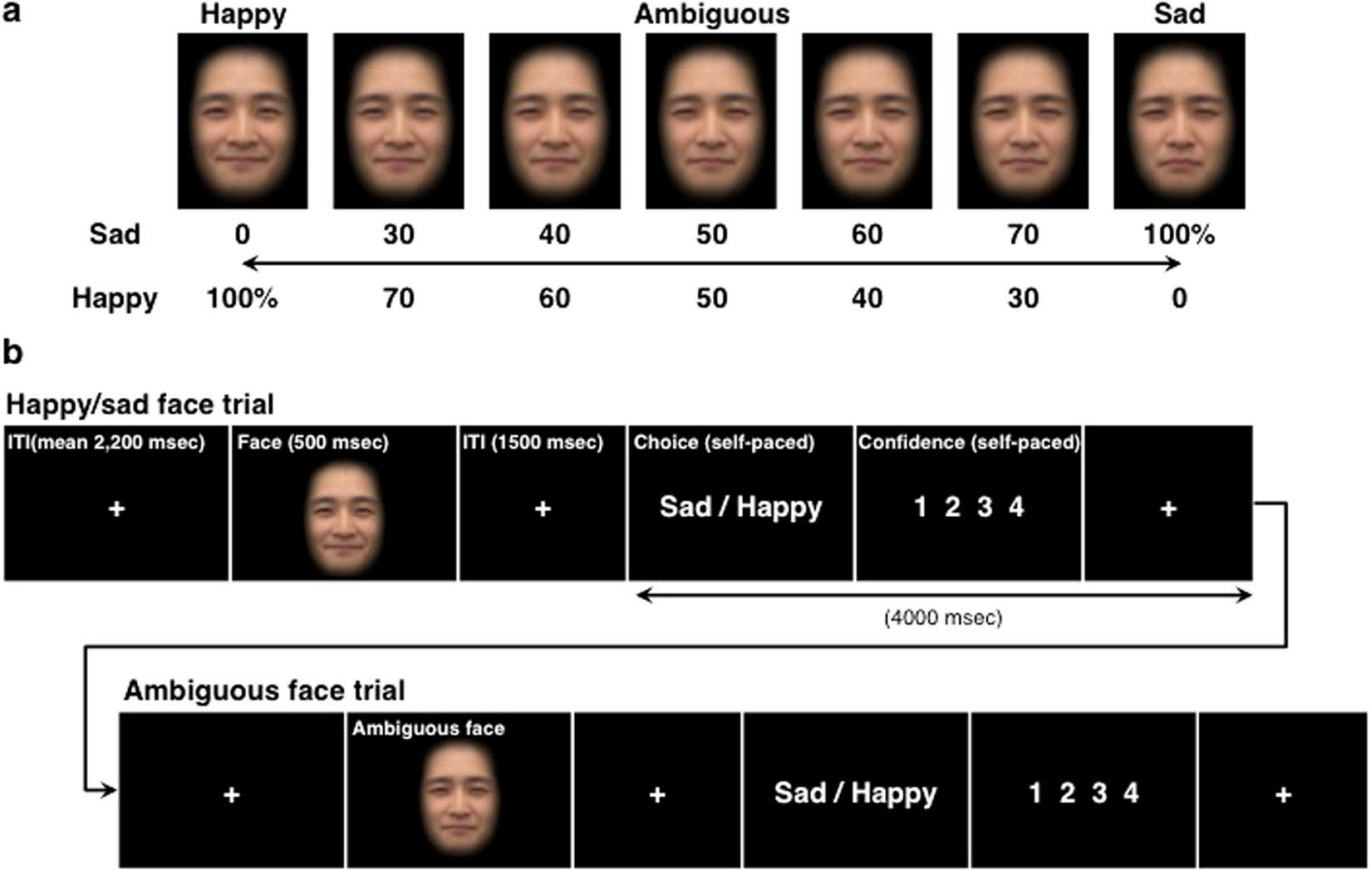
Face stimuli and the two-alternative forced choice task. (**a**) Representative example of the graded emotional facial expression stimuli used in the task, ranging from a 100% happy face to a 100% sad face. For the purpose of presentation, these example images were constructed by morphing the laboratory members and were not used in this experiment. (**b**) Two-alternative forced choice task. Ambiguous face trials were preceded by either “happy” or “sad” face trials. ITI: inter-trial interval.

The aim of the current study was to investigate the neuroanatomical correlates associated with negativity bias in response to ambiguous stimuli in healthy participants and to understand how the neural responses may be modified by the individual differences in hopelessness, which is a pessimistic characteristic of depression^30^. To explore these functional correlates, we used the two-alternative forced choice task involving a series of graded facial stimuli that morphed from happy to sad during event-related fMRI (Fig. 1). For each participant, we calculated the point of subjective equality (PSE), whereby a lower PSE indicates a larger negativity bias, and we used the PSE as a covariate of interest for fMRI data analysis to examine the neuroanatomical correlates of negativity bias.

## Results

### Behavioral results

**Figure 2:**
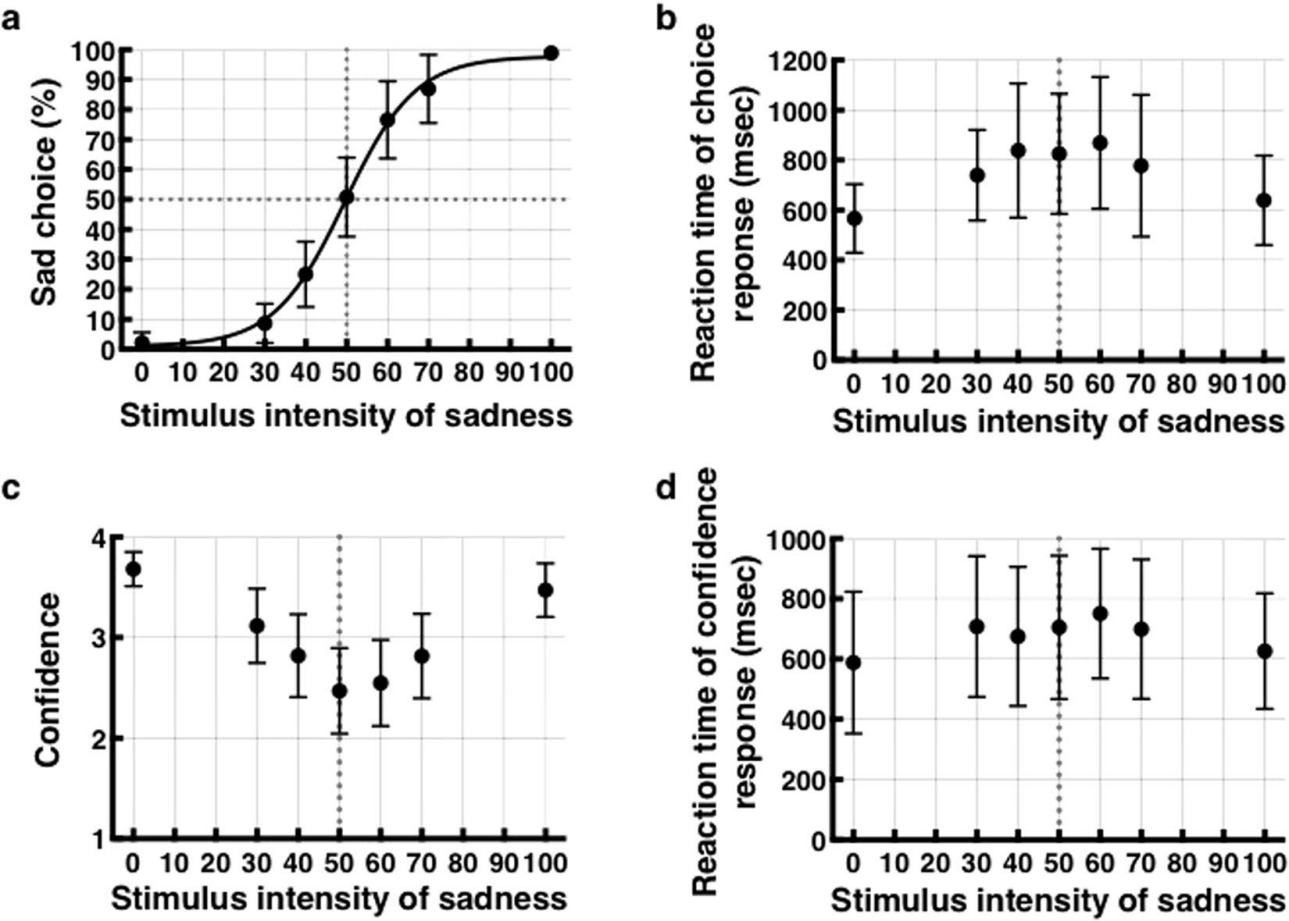
Behavioral data. (**a**) Behavioral choice patterns showing the rate of “sad” choices (vertical axis) as a function of the graded stimulus intensity of “sad” (horizontal axis). The fitted curve indicates the mean of all participants. (**b**) Reaction time for choice, (**c**) confidence rating, and (**d**) reaction time for confidence rating are shown. Error bars represent the standard deviation of the mean.

Task performance is shown in Fig. 2. The percentage of “sad” responses was near 0% for prototypical happy faces, was near 100% for prototypical sad faces, and exhibited a “sigmoidal” shape with 50% “sad” responses for ambiguous faces. The PSE of each participant was 50.78 ± 13.22% (mean and standard deviation (S.D.), range, 43.30–57.40%; Fig. 2a). Reaction times were fitted better with an inverted U-shaped curve (*p* = 0.004) than with linear and exponential equations (*p* = 0.633 and 0.594, respectively; Fig. 2b). The confidence level was also fitted best with a U-shaped curve (*p* = 0.011) compared to fitting with linear and exponential equations (*p* = 0.632 and 0.652, respectively; Fig. 2c). We also examined the correlation between the PSE and the Beck Hopelessness Scale (BHS^30^, mean, 8.07; median, 8.00; S.D., 4.17; range, 2–15; skewness, 0.132; kurtosis, −1.513 (Supplementary Table S1)), which was close to the average for the Japanese population (8.6)^31^. There was not a significant correlation between the PSE and the BHS.

### Imaging results

**Figure 3:**
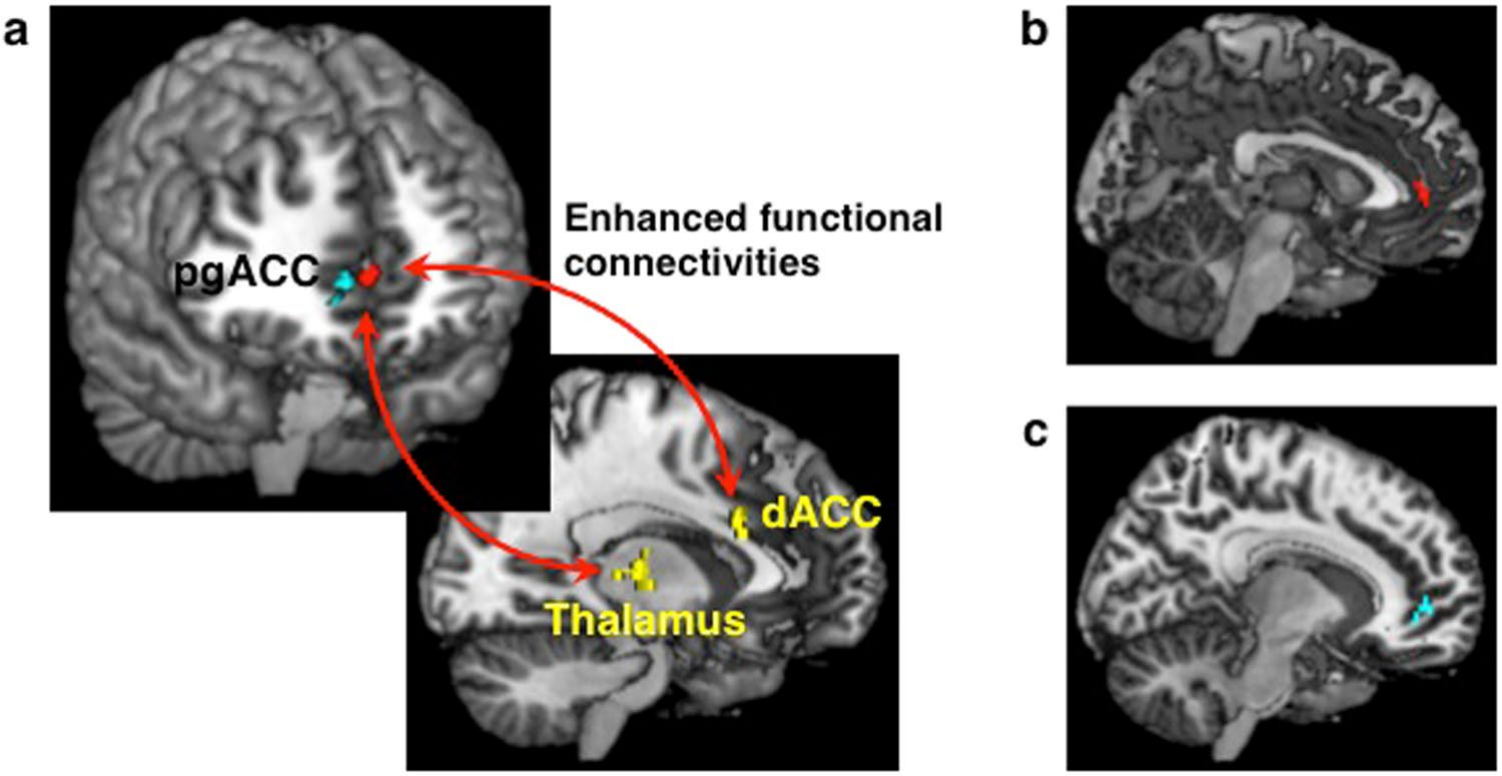
(**a**) Neural basis of negativity bias in facial perception. Activation of the bilateral pgACC (peak Montreal Neurological Institute coordinates: x, y, z = 0, 46, 4; Z = 3.55, red cluster in upper panel) during “sad” versus “happy” choices in response to ambiguous faces was negatively correlated with the point of subjective equality (PSE). The Beck Hopelessness Scale (BHS) score was positively correlated with functional connectivities of the bilateral pgACC with the right dACC and right thalamus (yellow clusters in lower panel). The right pgACC (x, y, z = 8, 46, 2; Z = 4.23, blue cluster in upper panel) exhibited a significant negative correlation with the PSE in the contrast of 100% “sad” versus 100% “happy” faces. Significant clusters in the left putamen and the left orbitofrontal cortex are not displayed. (**b**) Sagittal section showing the bilateral pgACC activity, which was negatively correlated with the PSE in the contrast of “sad” versus “happy” choices in response to ambiguous faces. This cluster was consistent with the red cluster in (**a**). (**c**) Sagittal section showing the right pgACC activity, which was negatively correlated with the PSE in the contrast of 100% “sad” versus 100% “happy” faces. This cluster was consistent with the blue cluster in (**a**).

Initially, we assessed activity associated with negativity bias during responses to ambiguous faces. For the purpose of this analysis, we divided the ambiguous face trials into two conditions based on whether the preceding trial was “sad” or “happy.” The fMRI data were analyzed based on participants’ “sad” versus “happy” choices using a general linear model, which included the PSE as a covariate of interest. Voxel-by-voxel statistical parametric mapping (SPM) analysis revealed that activation of the left putamen (peak Montreal Neurological Institute (MNI) coordinates: x, y, z = −26, 10, 8; Z = 3.91) and the bilateral pgACC (x, y, z = 0, 46, 4; Z = 3.55, red cluster in the upper panel of Fig. 3) was negatively correlated with the PSE for “sad” versus “happy” choices (Supplementary Table S2). Thus, the activities of the bilateral pgACC and of the left putamen were associated with a larger negativity bias. Based on 5,000 bootstrapped samples using bias-corrected and accelerated (BCa) 95% confidence intervals (CIs), there were significant correlations between the PSE and the neural activity of the left putamen (r = −0.82, *p* < 0.001, bias = 0.007, standard error (S.E.) = 0.083, BCa CI = (−0.92, −0.66)) and that of the bilateral pgACC (r = −0.78, *p* = 0.001, bias = 0.024, S.E. = 0.14, BCa CI = (−0.93, −0.43)) (Supplementary Figs S1 and S4). By contrast, there were no regions correlated with the PSE in the contrast of “happy” versus “sad.” The signal changes in the pgACC in “sad” and “happy” conditions are presented in Supplementary Fig. S7.

Next, to examine functional couplings with the bilateral pgACC, we conducted psychophysiological interaction (PPI) analysis^32^ based on a contrast of “sad” versus “happy.” Furthermore, the BHS score of each participant was included as a covariate of interest to examine the association between the functional couplings and this psychological feature of depression. PPI analysis revealed that the BHS score was positively correlated with the intensity of the functional connectivities of the bilateral pgACC with the right dorsal ACC (dACC) (x, y, z = 2, 18, 24; Z = 4.07) and right thalamus (x, y, z = 16, −18, 6; Z = 3.75) (yellow clusters in lower panel of Fig. 3, Supplementary Table S3). There were significant correlations between the BHS and the intensity of functional connectivity of the bilateral pgACC with the right dACC (r = 0.71, *p* = 0.003, bias = −0.012, S.E. = 0.137, BCa CI = (0.43, 0.88)) and with the right thalamus (r = 0.83, *p* < 0.001, bias = −0.009, S.E. = 0.089, BCa CI = (0.63, 0.94)) (Supplementary Figs S2 and S5). We also conducted PPI analysis including the PSE as a covariate of interest, but there were no regions correlated with the PSE.

Finally, we assessed the activity evoked while viewing 100% “sad” faces relative to 100% “happy” faces. The right pgACC (x, y, z = 8, 46, 2; Z = 4.23, blue cluster in upper panel of Fig. 3) and the left orbitofrontal cortex (OFC) (x, y, z = −2, 44, −10; Z = 3.83) each exhibited a significant negative correlation with the PSE in the contrast of 100% “sad” versus 100% “happy” faces (Supplementary Table S4), indicating that the activity of the right pgACC was also facilitated in a stimulus-driven manner in individuals with larger negativity bias. We found significant correlations between PSE and the neural activity of the right pgACC (r = −0.84, *p* < 0.001, bias = −0.007, S.E. = 0.063, BCa CI = (−0.93, −0.72)) and that of the left OFC (r = −0.90, *p* < 0.001, bias = 0.003, S.E. = 0.052, BCa CI = (−0.96, −0.80)) (Supplementary Figs S3 and S6).

## Discussion

The current study used a data-driven approach to examine the neural circuitry underlying negativity bias while viewing ambiguous faces. There were three main findings in this study. First, negativity bias for ambiguous facial expressions was positively associated with the bilateral pgACC activity. Second, the strength of the functional couplings between the bilateral pgACC and the right dACC/right thalamus were positively associated with hopelessness, which is a pessimistic characteristic of depression. Third, the right pgACC activity for the negativity bias of “sad” overlapped with activity induced by sad stimuli. Our findings identified the pgACC as the preferential site of activation for a negativity bias of sad over happy.

The two-alternative forced choice task and a graded facial stimulus series allowed us to calculate interindividual differences in negativity bias as the PSE. In addition, reaction time was longest and confidence was lowest for ambiguous faces, indicating that ambiguous faces were difficult to judge and confirming the concurrent perception of two opposing emotions.

The pgACC is thought to play a key role in emotion processing^33^. Activity of the pgACC has been associated with the assessment of emotional salience^33^ and attention to emotional information^34^ as well as with the regulation of affective conflicts in the context of emotional interference^35–37^. Moreover, the pgACC also plays a crucial role in the pathophysiology of depression^19^. Hyperactivity in the pgACC is related to increased neuroticism^38^ and mood-congruent sad processing^39^. This region also shows increased activity in depressive patients in the resting state^40^ and decreased activity in depressive patients in response to positive affective stimuli^41^. Moreover, late-life depression is the most frequent psychological disease and has been associated with cognitive impairment^42^ as well as pgACC hyperactivation^43^. Thus, hyperactivity of the bilateral pgACC may represent a latent cognitive trait of depression in the healthy individuals. The pgACC has also been implicated in social cognitive functions^33, 44^, such as self-reflection^45^, person knowledge^46^ and mentalizing^47^. Taken together, the current findings may suggest that a negativity bias has impacts on the social functioning of hopeless, or pessimistic, people.

The dACC is part of the salience network, which functions to identify the most relevant of several internal and external stimuli to guide appropriate behavior^48^, and it also participates in conflict processing^49, 50^ and decision making. In addition to cognitive functions, the dACC is involved in emotional processing. For instance, a clinical study indicated that lesions of the dACC altered emotional experience^51^, and meta-analytic neuroimaging data showed that dACC activity was also associated with emotional processing^52^. Taken together, through the stronger functional coupling between the pgACC and the dACC, ambiguous emotion perception may be resolved by the biased decision-making toward the salient “sad” information in individuals who possess a depressive trait of hopelessness.

Finally, the thalamus is classically considered to be the sensory relay region that regulates the transmission of information to the cortex and between cortical regions, including for perception and cognition^53^. Thus, the thalamus is an important region for vigilance^54, 55^ and is an integral part of the emotional and affective circuit^52, 56, 57^. Overactivation of the thalamus has been reported in depression^5, 58^. The pgACC and dACC modulate the thalamus-amygdala relationship^59–61^. The thalamus has also been considered a relevant subcortical structure in fear circuitry^62^ and has been included in the core limbic region of emotion-processing networks, mediating further signal transmission and interactions between subcortical areas and cortical areas^63^. Given these various roles of the thalamus, neural circuits of the pgACC, dACC, and thalamus may be important for directing attention to “sadness” as salient information in ambiguous perceptual situations in people with higher hopelessness.

The amygdala has been strongly associated with the processing of emotionally arousing stimuli, and most of the relevant neuroimaging studies show abnormalities in depressive patients in the face-processing network, indicating a mood-congruent bias of hyperactivation of the amygdala to negative stimuli^25, 56, 64^. Indeed, the activity of the amygdala exhibited a negative correlation with the PSE in the current study (*p* = 0.001 (uncorrected), k = 11, Supplementary Fig. S8). Thus, the activity of the amygdala was also positively associated with the negativity bias in healthy individuals. In addition, previous studies have shown that the amygdala was involved in coding facial expressions of sadness^65^ but also of happiness^66^, and the current finding using the direct comparison between sadness and happiness may favor the former.

There are several limitations to this study. First, our study included only male participants to avoid potentially confounding gender effects on the processing of emotional stimuli^67^. Future studies should explore possible gender differences in relation to the findings of the current study. Second, we studied only healthy participants. As negativity bias is a characteristic of depression, this study should be replicated in patients with depression to determine the relationship between depressive symptoms and our proposed neuroanatomical correlates of negativity bias. Third, the sample size of this study was relatively small; therefore, the study might be underpowered^68^. To compensate for the small sample size, we conducted a resampling procedure with 5,000 bootstrapped samples. It is possible that a larger study would find more variation in the neural basis of negativity bias. Fourth, we used the BHS to measure the depressive trait of hopelessness, but contrary to our expectations, the BHS was not correlated with PSE. We cannot explain this discrepancy, but the current findings may suggest that the BHS and the PSE are mediated by a specific neural function. Further study is needed to clarify this issue.

In summary, negativity bias was associated with hyperactivity in the bilateral pgACC, which has been related to the depressive trait of hopelessness^69^. Components of the salience network, such as the right dACC and the right thalamus, exhibited positive functional couplings with the bilateral pgACC, suggesting a negative over a positive perception of ambiguous facial expressions in individuals with higher hopelessness. These regional associations may underlie the negativity bias in depression, which may influence both symptom formation and social dysfunction. The current finding that the pgACC is a potential neuroanatomical correlate of negativity bias suggests that this region may be an appropriate target for neurofeedback techniques that aim to reduce negativity bias and enhance positive perceptions of the outside world; such neurofeedback techniques targeting the pgACC will certainly be the focus of future studies in depressed patients.

## Methods

### Participants

Nineteen right-handed healthy male volunteers (mean age, 24.1 ± 4.1 (S.D.) years) were recruited for this study. Four volunteers were removed from all analyses due to anatomical abnormalities, e.g., enlarged perivascular space or white matter lesions. Data from fifteen volunteers (mean age, 24.5 ± 4.5 years; range, 20–34) were included in the final analysis. These participants had no history of neurological or psychiatric disorders and were not taking any medications that could interfere with task performance or fMRI data. All participants provided written informed consent before participating in the study, which was approved by the Ethics and Radiation Safety Committee of the National Institute of Radiological Sciences in accordance with the ethical standards laid down in the 1964 Declaration of Helsinki and its later amendments.

### Stimuli and behavioral task

Identity-matched faces with happy and sad expressions (eight identities) were obtained from the ATR Facial Expression Image Database (DB99, Advanced Telecommunications Research Institute International). The image contrast and luminance of the face images were normalized using Adobe Photoshop CC (Adobe Systems, Inc. California, USA). For each identity, a complete graded stimulus series was generated by morphing faces with happy and sad expressions (Fig. 1a). Each graded stimulus series included a total of seven intensities, starting with a 100% “happy” face (i.e., 0% “sad” face) and ending with a 100% “sad” face. Five intermediate intensities between “happy” and “sad” were generated (30%, 40%, 50% (ambiguous), 60%, and 70% “sad”) using the morphing technique in the Abrosoft FantaMorph software package (Beijing, China). Henceforth, “happy” refers to faces with 0%, 30%, or 40% morphing to a sad expression, and “sad” refers to faces with 60%, 70%, or 100% morphing to a sad expression. A total of 56 stimuli were used in the study (eight identities x seven intensities).

Participants performed a two-alternative forced choice task in which they were asked to judge whether the expression of a briefly presented face was either sad or happy (Fig. 1a). The viewing distance was 84.5 cm, and each face image had an overall size of angle (8.8 height × 8.2 width degrees). Participants were instructed that some of the faces would have ambiguous expressions. Participants were encouraged to be accurate in their judgments but to respond reasonably quickly. In an event-related design, each trial began with a fixation cross shown on a black screen for a jittered duration (mean duration, 2,200 ms; range, 1,400–4,200 ms), followed by the presentation of a face for 500 ms and then another fixation cross for 1,500 ms. Participants were then asked to judge the facial expression as either “happy” or “sad” via button press. Button assignment was counterbalanced across participants (seven participants used the index finger and middle finger for sad and happy responses, respectively; reverse mapping was used for the other eight participants). Following the emotion identification phase, participants were asked to rate their level of confidence in their decision using a four-point scale ranging from 1 (low confidence) to 4 (high confidence) to confirm whether the ambiguous faces were perceived as showing ambiguous facial expressions. If the ambiguous faces were indeed perceived as ambiguous, the confidence for the ambiguous faces would be the lowest among all faces. The 48 non-ambiguous stimuli were presented three times each, whereas the eight ambiguous stimuli were presented six times each. Altogether, the participants performed 288 trials (144 trials for ambiguous stimuli) across six fMRI scans. In general, ambiguous face trials were preceded by “happy” or “sad” face trials. Outside the scanner, all participants completed the Japanese version of the Beck Hopelessness Scale (BHS)^30^.

### Behavioral data analysis

The response pattern of each participant was fitted with a sigmoidal Weibull function (equation 1), which was defined by two parameters: the point of subjective equality (PSE) α, and slope β^70^. Y represents the percentage of “sad” choices, and X represents the stimulus intensity of “sadness”. The quality of fit for each participant was assessed by correlating predicted values from the best-fitting psychometric function with the observed accuracy (*R* ^*2*^ for group = 0.989 ± 0.013 (S.D.), range, 0.945–0.998). The overall response pattern of participants is shown in Fig. 2a (α = 50.78 ± 13.22%, range, 43.30–57.40%; β = 0.0052 ± 0.0020, range, 0.0021–0.0090). Analysis of the psychometric function was performed using GraphPad Prism version 6.00 for Mac (GraphPad Software, San Diego, CA, USA).

1 $$Y=100/(1+{10}^{(\alpha -X)\times \beta })$$

Polynomial curve-fitting procedures (i.e., linear, exponential) to test the association between the stimulus intensity of “sadness” and the mean reaction time and self-reported confidence were performed in SPSS (IBM Corp. Released 2012. IBM SPSS Statistics for Macintosh, Version 21.0. Armonk, NY: IBM Corp.).

### fMRI procedures

#### Data acquisition

The two-alternative forced choice task was presented using E-Prime 2.0 software (Psychology Software Tools, Pittsburgh, PA, USA). A Siemens Verio MRI system (3T) was used to obtain T2*-weighted echo-planar imaging (EPI; repetition time [TR] = 2,000 ms, echo time [TE] = 25 ms, slice number = 33, thickness = 3.8 mm, matrices = 64 × 64, 209 volumes [lead in 6 volumes, lead out 3 volumes], interleaved acquisition) and structural T1 images (TR = 2,300 ms, TE = 1.95 ms, slice number = 176, thickness = 1 mm, matrices = 256 × 256).

### fMRI preprocessing

fMRI images were analyzed using Statistical Parametric Mapping (SPM8; www.fil.ion.ucl.ac.uk/spm). EPI data were corrected for slice timing, rigid head motion, and susceptibility artifacts (“realign and unwarp”). Head motion parameters were examined, and we confirmed that all trials had less than one voxel of translation and 1.5° of rotation in each participant. Then, individual structural T1 images were co-registered to the mean functional image that was generated during realignment. Co-registered T1 images were segmented using the “new segment” routine in SPM8. Tissue-class images for gray and white matter were generated and used within the DARTEL toolbox in SPM8 to create structural templates as well as individual flow fields, which were used for normalization to the MNI space. Data were smoothed using a 6-mm full-width at half maximum (FWHM) isotropic Gaussian kernel.

### fMRI first-level analysis

A two-level analysis was performed in SPM8. At the single-subject level, the onset times for each face in all trials were modeled as separate regressors by convolving stimulus onsets with a canonical hemodynamic response function. As reported previously^71, 72^, the facial perception was affected by the preceding facial expression. According to the adaptation theory, a preceding happy face makes the subsequent ambiguous face look sad, and a preceding sad face makes the subsequent ambiguous face look happy, and we only included the successive trials where a “sad” response to an ambiguous face followed a happy face or a “happy” response to an ambiguous face followed a happy face. Thus, to examine negativity bias in the perception of ambiguous faces, ambiguous face trials were divided into two conditions based on participant perception: a “sad” choice for an ambiguous face after a “happy” face trial (average number of trials: 37.60 ± 10.30) and a “happy” choice for an ambiguous face after a “sad” face trial (36.47 ± 11.06). Overall, eight regressors were created based on the “happy,” “sad,” and ambiguous face stimuli. Six realignment parameters (head motion correction) and two derivatives were used as covariates. All artifacts in fMRI time-series data were detected and corrected using RobustWLS in SPM8^73^.

### fMRI second-level analysis

Individual contrasts were analyzed in a random-effects model, and the contrasts were constructed using T-contrasts. The contrast of “sad” versus “happy” choices in response to ambiguous faces was analyzed to examine the neural correlates of negative emotional perception in an ambiguous situation. We also analyzed the contrast of 100% “sad” versus 100% “happy” faces for comparison. The PSE of each participant, which was mean-centered, was included as a covariate of interest to assess the effect of negativity bias on these two contrasts. Age was mean-centered and included as a nuisance covariate. Local maxima with *p* < 0.001 (uncorrected) and an extent threshold of 20 voxels were considered to be statistically significant^74^. To compensate for our small sample size, we used a resampling procedure based on 5,000 bootstrapped samples using bias-corrected and accelerated (BCa) 95% confidence intervals (CIs) to estimate Pearson’s correlation coefficient between the neural activities and the PSE scores. Each region of interest (ROI) was analyzed using the MarsBar tool for SPM (http://marsbar.sourceforge.net/). For extracting ROI data, we used the cluster image of each region.

### Psychophysiological interaction analysis

One established method for characterizing functional connectivity within brain networks in the context of experimental tasks is psychophysiological interaction (PPI) analysis^32^. A series of PPI analyses were carried out in SPM8 to capture the influence of our task (the psychological factor) on the strength of the functional coupling (functional connectivity) between brain regions in relation to the experimental design. To conduct the PPI analysis, we extracted the deconvolved time courses from the left pregenual ACC of each participant (based on the red cluster in Fig. 3). We then calculated the product of the deconvolved activation time course and the vector of the psychological variable of interest (“sad” versus “happy” choice in response to an ambiguous face) to create the PPI term. Individual-level PPIs were computed for each participant and then entered into a second-level random-effects analysis. For second-level analysis, the BHS and PSE scores of each participant, which were mean-centered, were included as covariates of interest to examine the correlation between functional couplings and personal traits. Age was mean-centered and included as a nuisance covariate. Local maxima with *p* < 0.001 (uncorrected) and an extent threshold of 20 voxels were considered to be statistically significant^74^. We used a resampling procedure based on 5,000 bootstrapped samples using BCa CI for correlational analysis between the intensity of functional connectivity and the BHS scores. For extracting ROI data, we used the cluster image of each region.

## Electronic supplementary material

Supplementary Information
