# Supplementary material for: Neural basis of negativity bias in the perception of ambiguous facial expression
Source: Sci Rep. 2017 Mar 24;7:420. doi: 10.1038/s41598-017-00502-3 (PMC5428736; doi:10.1038/s41598-017-00502-3)
Supplement: Supplementary file 1 — Supplementary Information [file 41598_2017_502_MOESM1_ESM.doc]

**Supplementary Information**

**Title:** Neural basis of negativity bias in the perception of ambiguous facial expression

**Authors:**

Takehito Ito, Keita Yokokawa, Noriaki Yahata, Ayako Isato Tetsuya Suhara, Makiko Yamada

**Supplementary Figure S1.** Negative correlations between the point of subjective equality (PSE) and the neural activity of the left putamen based on 5,000 bootstrapped samples using bias-corrected and accelerated (BCa) 95% confidence intervals (CIs) (Pearson’s correlation coefficient r = -0.82, *p* < 0.001, bias = 0.007, standard error (S.E.) = 0.083, BCa CI = (-0.92, -0.66)) and that of the bilateral pregenual anterior cingulate cortex (pgACC) (r = -0.78, *p* = 0.001, bias = 0.024, S.E. = 0.137, BCa CI = (-0.93, -0.43). The dashed lines indicate 95% confidence intervals.

**Supplementary Figure S2.** Positive correlations between the Beck Hopelessness Scale (BHS) and the intensity of the bilateral pgACC functional connectivity of the right dorsal ACC (dACC) based on 5,000 bootstrapped samples using BCa CI (r = 0.71, *p* = 0.003, bias = -0.012, S.E. = 0.137, BCa CI = (0.43, 0.88)) and that with the right thalamus (r = 0.83, *p* < 0.001, bias = -0.009, S.E. = 0.089, BCa CI = (0.63, 0.94)). The dashed lines indicate 95% confidence intervals.

**Supplementary Figure S3.** Negative correlations between the PSE and the activity of the right pgACC based on 5,000 bootstrapped samples using BCa CI (r = -0.84, *p* < 0.001, bias = -0.007, S.E. = 0.063, BCa CI = (-0.93, -0.72)) and that of the left OFC (r = -0.90, *p* < 0.001, bias = 0.003, S.E. = 0.052, BCa CI = (-0.96, -0.80)). The dashed lines indicate 95% confidence intervals.

**Supplementary Figure S4.** Exploratory overview of brain activations in the contrast of “sad” versus “happy” for ambiguous faces. Scatter plots show the average and standard deviation (S.D.). Each dot shows the value for an individual participant. Also see Supplementary Table S2.

**Supplementary Figure S5.** Exploratory overview of the intensities of functional connectivities with the pgACC. Scatter plots show the average and S.D. Each dot shows the value for an individual participant. Also see Supplementary Table S3.

**Supplementary Figure S6.** Exploratory overview of brain activations in the contrast of 100% sad face versus 100% happy face. Scatter plots show the average and S.D. Each dot shows the value for an individual participant. Also see Supplementary Table S4.

**Supplementary Figure S7.** Brain activations of each participant in the contrast of “sad” and “happy” for ambiguous faces. Scatter plots show the average and standard deviation (S.D.). Each dot represents the value for an individual participant.

**Supplementary Figure S8.** Activation of the left amygdala (x, y, z =-24, -2, -14; Z = 3.37, k = 11, red cluster) during “sad” versus “happy” choices in response to ambiguous faces was negatively correlated with the PSE.

**Supplementary Table S1.** Subject information.

**Supplementary Table S2.** Brain regions that were negatively correlated with the PSE in the contrast of “sad” versus “happy” for ambiguous faces.

**Supplementary Table S3.** Brain regions that were positively correlated with the BHS in PPI analysis.

**Supplementary Table S4.** Brain regions that were negatively correlated with the point of psychological equality in the contrast of 100% sad face vs. 100% happy face.
